# Efficacy, Safety, and Survival Outcomes of Immune Checkpoint Inhibitors in Patients with Mismatch Repair-Deficient Colorectal Cancer: A Retrospective, Multicenter Study

**DOI:** 10.3390/jcm15041554

**Published:** 2026-02-16

**Authors:** Mehmet Cihan İcli, Deniz Can Guven, Arif Akyildiz, Ali Fuat Gürbüz, Nargiz Majidova, Mehmet Mutlu Kıdı, Hakan Kosku, Elif Sahin, Tugce Kubra Gunes, Mustafa Seyyar, Elvina Almuradova, Pervin Can Sancı, Burak Bilgin, Ismail Oguz Kara, Mehmet Artac, Ömer Dizdar, Suayib Yalcin

**Affiliations:** 1 Department of Medical Oncology, Hacettepe University, Ankara 06100, Türkiye; denizcguven@hotmail.com (D.C.G.); drakyildizarif@gmail.com (A.A.); dromerdizdar@gmail.com (Ö.D.); suayibyalcin@gmail.com (S.Y.); 2 Department of Oncology, Erzincan Mengücek Gazi Training and Research Hospital, Erzincan 24100, Türkiye; 3 Department of Oncology, Necmettin Erbakan University, Konya 42090, Türkiye; dr.alifuatg@gmail.com (A.F.G.); zaratarum@eyahoo.com (M.A.); 4 Department of Oncology, VM Medical Park Maltepe Hospital, Istanbul 34846, Türkiye; nergiz.mecidova1991@gmail.com; 5 Department of Oncology, Cukurova University, Adana 01330, Türkiye; mehmetmutlu01@hotmail.com (M.M.K.); iokara@cu.edu.tr (I.O.K.); 6 Department of Oncology, Bilkent City Hospital, Ankara 06800, Türkiye; hakankosku@gmail.com (H.K.); drbbilgin@hotmail.com (B.B.); 7 Department of Oncology, Kocaeli City Hospital, Kocaeli 41060, Türkiye; dr_elifsahin48@hotmail.com; 8 Department of Oncology, Umraniye Training and Research Hospital, Istanbul 34764, Türkiye; drtugcekubragunes@gmail.com; 9 Department of Oncology, Gaziantep City Hospital, Gaziantep 27470, Türkiye; mustafaseyyar27@hotmail.com; 10 Department of Oncology, Memorial Goztepe Hospital, Istanbul 34634, Türkiye; almuradovaelvina@gmail.com; 11 Bakırçay University Çiğli Training and Research Hospital, İzmir 35620, Türkiye; ppervincan@hotmail.com

**Keywords:** MSI-H colorectal cancer 1, immunotherapy 2, overall survival 3, progression-free survival

## Abstract

**Background**: Microsatellite instability-high (MSI-H) or mismatch repair-deficient (dMMR) colorectal cancer (CRC) accounts for approximately 5% of metastatic CRC cases. Immune checkpoint inhibitors (ICIs) are the standard of care based on pivotal clinical trials; however, real-world data, particularly from low-resource countries, remain scarce, and prognostic factors are not yet fully defined. Therefore, we evaluated the efficacy and the safety of ICIs in a multi-center cohort. **Methods**: This multi-center retrospective study included 45 patients treated with ICIs across six oncology centers in Türkiye between June 2017 and December 2024. Patients received either anti–PD-1/PD-L1 monotherapy or anti–CTLA-4–based combination therapy. Key clinical variables and 1-, 2-, and 3-year OS and PFS outcomes were systematically collected. **Results**: The median age was 61 years, and most patients (75.6%) received ICIs in later treatment lines. After a median follow-up of 24.1 months, median OS and PFS were not reached. The estimated 1-, 2-, and 3-year OS rates were 82%, 76.1%, and 76.1%; PFS rates were 75.6%, 67.5%, and 67.5%, respectively. In multivariate analysis, an ECOG < 1 (HR: 0.072; 95% CI: 0.012–0.453; *p* = 0.005), a metastatic burden of fewer than two sites (HR: 0.211; 95% CI: 0.052–0.860; *p* = 0.030), and absence of antibiotic exposure within one month prior to immunotherapy initiation (HR: 0.145; 95% CI: 0.034–0.614; *p* = 0.009) were independently associated with improved overall survival. For PFS, ECOG < 1 (HR: 0.172; 95% CI: 0.052–0.573; *p* = 0.004), a metastatic burden of fewer than two sites (HR: 0.248; 95% CI: 0.078–0.788; *p* = 0.018), and no recent antibiotic exposure (HR: 0.209; 95% CI: 0.064–0.687; *p* = 0.010) remained independent predictors of prolonged survival. **Conclusions**: We observed overall survival outcomes similar to those reported in phase III clinical trials of immunotherapy in MSI-H/dMMR colorectal cancer, despite a substantial proportion of patients receiving immunotherapy in later lines of treatment. These findings support immune checkpoint inhibitors as the standard of care for MSI-H/dMMR metastatic colorectal cancer and emphasize the importance of improving access to immunotherapy, particularly in low-resource settings.

## 1. Introduction

Immunotherapy has become a central paradigm in modern cancer care, fundamentally reshaping treatment strategies and survival outcomes over the past decade [1,2,3]. ICIs restore antitumor immune surveillance by blocking inhibitory signaling through CTLA-4 and PD-1/PD-L1 pathways, leading to durable and clinically meaningful benefit across highly immunogenic tumors such as melanoma, non–small cell lung cancer, and renal cell carcinoma [4,5].

MSI-H colorectal cancers, which correspond to approximately 5% of patients with mCRC, represent a prototypical immune-hot subtype, characterized by frameshift mutation–driven neoantigen enrichment. Clinical trials have consistently shown that ICIs achieve high response rates and prolonged survival in this population [4,5,6]. Recent clinical trials have demonstrated durable responses and significant survival benefits with anti–PD-1– and anti–CTLA-4–based immunotherapy, establishing ICIs as the standard first-line treatment for patients with MSI-H/dMMR mCRC [7,8,9]. However, these trials predominantly enrolled highly selected patients with favorable performance status. In real-world settings, where patients often present with more heterogeneous clinical profiles, the efficacy and tolerability of ICIs remain less clearly defined [10,11]. Furthermore, in resource-limited settings like low-middle income countries, ICI access is not available in the first-line setting, further limiting the generability of clinical trial data with ICIs to the real world. To date, real-world evidence assessing the efficacy and safety profile of immunotherapy in patients with MSI-H/dMMR mCRC remains limited [9]. Yet, the management of this molecular subgroup has undergone a rapid and profound paradigm shift in recent years. Accordingly, objectively evaluating the contribution of ICIs in routine clinical practice, while identifying predictive factors associated with long-term benefit, is crucial for optimizing clinical decision-making [7,12].

Despite the strong efficacy signals observed in clinical trials, uncertainty remains regarding immunotherapy outcomes in routine practice, particularly among heterogeneous patient populations and in low- and middle-income countries. Real-world data are therefore essential to contextualize trial findings and inform treatment strategies across diverse healthcare settings [13].

In this multi-center retrospective study, we analyzed data from patients with MSI-H/dMMR mCRC treated with immunotherapy across six oncology centers in Türkiye to evaluate its real-world efficacy, safety, and survival outcomes, and to explore clinical factors associated with sustained ICI benefit.

## 2. Materials and Methods

### 2.1. Patients

This retrospective observational study included a total of 45 patients diagnosed with advanced MSI-H or dMMR metastatic colorectal cancer who received immunotherapy between June 2017 and December 2024 across six oncology centers in Türkiye. MSI-H/dMMR status was determined using immunohistochemistry (IHC) for mismatch repair proteins and/or polymerase chain reaction (PCR)-based microsatellite instability testing, and/or next-generation sequencing (NGS), according to institutional standards. All patients were treated with either anti–PD-1/PD-L1 monotherapy or combination regimens incorporating anti–CTLA-4 agents in routine clinical practice and at the discretion of the treating physician. Demographic and clinical data were retrieved from patient files and electronic hospital registries using a standardized data collection form. Variables assessed included age, sex, Eastern Cooperative Oncology Group (ECOG) performance status, presence of liver metastases, number of metastatic sites, line of immunotherapy, antibiotic use within the 30 days prior to initiation of immunotherapy, type of immunotherapy regimen administered, and duration of follow-up.

#### Patient Selection: Inclusion and Exclusion Criteria

Inclusion criteria:

- Age ≥ 18 years
- Histologically confirmed metastatic colorectal adenocarcinoma
- Microsatellite instability–high (MSI-H) or mismatch repair–deficient (dMMR) status confirmed by immunohistochemistry (IHC), polymerase chain reaction (PCR), next-generation sequencing (NGS), or a combination of these methods
- Treatment with at least one dose of immune checkpoint inhibitor therapy (anti–PD-1/PD-L1 monotherapy or anti–CTLA-4 based combination regimens)
- Treatment administered between June 2017 and December 2024 at one of the six participating oncology centers in Türkiye
- Availability of baseline clinicopathological data and follow-up information

Exclusion criteria:

- Microsatellite stable (MSS) or mismatch repair–proficient colorectal cancer
- Non-adenocarcinoma histology
- Patients who did not receive immune checkpoint inhibitors
- Insufficient clinical or follow-up data
- Age < 18 years

### 2.2. Endpoints

The primary endpoints were overall survival (OS) and progression-free survival (PFS). OS was defined as the time from initiation of immune checkpoint inhibitor (ICI) therapy to death from any cause; patients alive at the last follow-up were censored at that date. PFS was defined as the time from treatment initiation to radiologically confirmed disease progression or death, whichever occurred first; patients alive without documented progression were censored at the date of last follow-up. Tumor response and disease progression were assessed according to RECIST version 1.1 based on routine radiology reports obtained from clinical records. No cases were considered to represent pseudoprogression during follow-up.

Secondary endpoints included the objective response rate (ORR), the proportion of patients with a complete or partial response, and the disease control rate (DCR), the proportion with a complete or partial response or stable disease as the best overall response. Hyperprogressive disease (HPD) was defined as a marked acceleration in tumor growth kinetics during ICI therapy, characterized by at least a twofold increase compared with the pre-treatment period and radiologically confirmed progression within the first 6–8 weeks of treatment, as previously defined [14]. Patients who completed 2 years of immunotherapy without disease progression continued treatment beyond 2 years according to the treating physician’s clinical judgment and institutional practice.

### 2.3. Statistical Analysis

For OS and PFS, the duration of follow-up was estimated using the reverse Kaplan–Meier method. Univariate survival analyses were performed using Kaplan–Meier estimates, and differences between prognostic groups were assessed with the log-rank test. A multivariable Cox regression model with backwards variable selection was constructed for OS and PFS and variables with a *p*-value ≤ 0.10 in univariate analysis were included in the multivariable model. Hazard ratios (HRs) and corresponding 95% confidence intervals (CIs) were reported. All statistical analyses were performed using SPSS software, version 24.0 (IBM Corp. Armonk, NY, USA). A two-sided *p*-value < 0.05 was considered statistically significant.

## 3. Results

### 3.1. Baseline Characteristics of Patients

A total of 45 patients with MSI-H/dMMR mCRC were included in the analysis. The median age at treatment initiation was 61 years (range, 21–78), with 26 patients (57.8%) aged < 65 years. The majority were male (N = 33, 73.3%). A total of 62.2% of the patients had an ECOG performance status of 0. The primary tumor site was the right colon in most patients (64.4%). Liver metastases were present in 28 patients (60.0%), and lung metastases were present in 13 (28.9%). A family history of colorectal cancer was documented in 8 patients (17.8%), absent in 34 (75.6%), and unknown in 3 (6.7%). MSI status was determined by immunohistochemistry (IHC) in over 80% of the patients (86.7%). BRAF mutations were detected in 12 patients (26.7%) and wild-type BRAF in 29 (64.4%). 33 patients (73.3%) were RAS wild-type. Seven patients (15.6%) had received antibiotics within 1 month prior to initiation of ICI therapy. The most frequently administered ICI regimen was pembrolizumab (N = 33, 73.3%), followed by nivolumab (N = 7, 15.6%) and the combination of nivolumab plus ipilimumab (N = 5, 11.1%). Baseline patient and disease characteristics are summarized in Table 1.

### 3.2. Survival Outcomes with Immune Checkpoint Inhibitors

**Figure 1:**
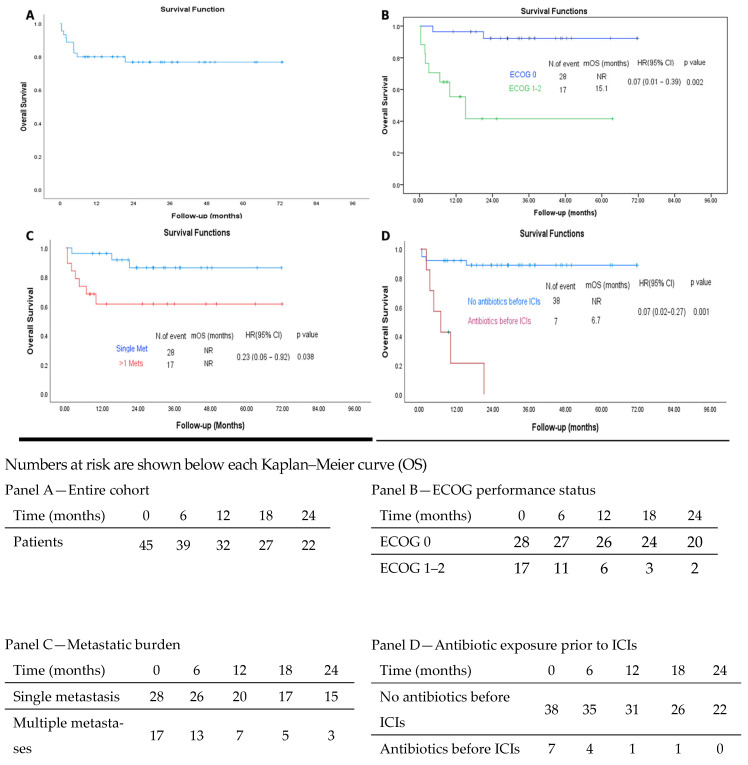
Overall survival (OS) analyses in patients with MSI-H/dMMR metastatic colorectal cancer treated with immune checkpoint inhibitors (ICIs). (**A**) Kaplan–Meier curve for OS in the overall study population. (**B**) OS stratified by ECOG-PS (ECOG 0 vs. ECOG 1–2). (**C**) OS according to metastatic burden (single vs. multiple metastases). (**D**) OS according to antibiotic exposure prior to initiation of ICIs. Abbreviations: mOS, median overall survival; ICIs, immune checkpoint inhibitor; ECOG Eastern Cooperative Oncology Group; Met, metastasis; Mets, metastases; NR, not reached; HR, hazard ratio; CI, confidence interval.

**Figure 2:**
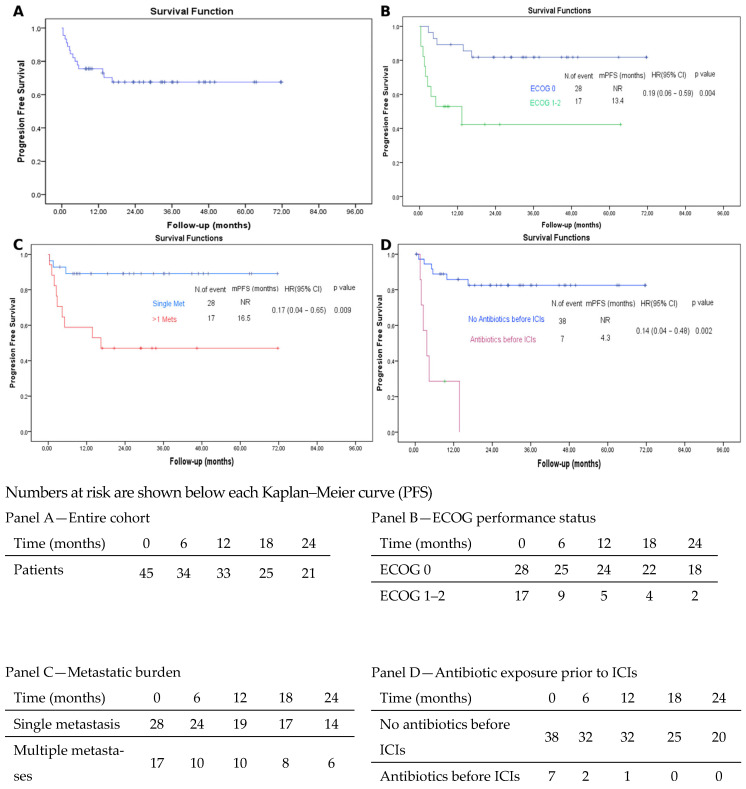
Progression-free survival (PFS) analyses in patients with MSI-H/dMMR metastatic colorectal cancer treated with immune checkpoint inhibitors (ICIs). (**A**) Kaplan–Meier curve for PFS in the overall study population. (**B**) PFS stratified by ECOG-PS (ECOG 0 vs. ECOG 1–2). (**C**) PFS according to metastatic burden (single vs. multiple metastases). (**D**) PFS according to antibiotic exposure prior to initiation of ICIs. Abbreviations: mPFS, median progression-free survival; ICIs, immune checkpoint inhibitor; ECOG-, Eastern Cooperative Oncology Group performance status; Met, metastasis; Mets, metastases; NR, not reached; HR, hazard ratio; CI, confidence interval.

The median follow-up for the entire cohort was 26.7 months (range, 0.39–71.75). At the data cut-off, 10 patients (22.2%) had died and 35 (77.8%) were alive. Median OS was not reached. The estimated survival rates at 1, 2, and 3 years were 82.0% (95% CI, 70.6–93.4), 76.1% (95% CI, 63.0–89.2), and 76.1% (95% CI, 63.0–89.2), respectively (Figure 1). Similarly, median PFS was not reached. The estimated PFS rates at 1, 2, and 3 years were 75.6% (95% CI, 63.1–88.1), 67.5% (95% CI, 53.4–81.6), and 67.5% (95% CI, 53.4–81.6), respectively (Figure 2). The best response to immunotherapy was complete response (CR) in 15.6% of patients, partial response (PR) in 44.4%, stable disease (SD) in 11.1%, and progressive disease (PD) in 24.4% of the cohort. Among patients with PD, 18.2% (n = 2) fulfilled the predefined criteria for hyperprogressive disease (HPD), corresponding to 4.4% of the entire study population. The objective response rate (ORR) was 60.0%, while the disease control rate (DCR) reached 71.1% (Table 2).

#### 3.2.1. Predictors of OS

In univariate analysis, an ECOG performance status < 1 (*p* = 0.002), a metastatic burden of fewer than two sites (*p* = 0.038), absence of recent antibiotic exposure (*p* < 0.001), and a borderline association with RAS wild-type status (*p* = 0.048) were associated with improved OS. In the multivariate Cox regression model, an ECOG performance status < 1 (HR: 0.072; 95% CI: 0.012–0.453; *p* = 0.005), a metastatic burden of fewer than two sites (HR: 0.211; 95% CI: 0.052–0.860; *p* = 0.030), and absence of antibiotic exposure within one month prior to immunotherapy initiation (HR: 0.145; 95% CI: 0.034–0.614; *p* = 0.009) were independently associated with improved OS (Table 3).

#### 3.2.2. Predictors of PFS

In univariate analysis, an ECOG performance status < 1 (*p* = 0.004), a metastatic burden of fewer than two sites (*p* = 0.027), absence of antibiotic exposure within one month prior to immune checkpoint inhibitor initiation (*p* = 0.001), and RAS wild-type status (*p* = 0.034) were significantly associated with improved PFS. In the multivariate Cox regression model, an ECOG performance status < 1 (HR: 0.172; 95% CI: 0.052–0.573; *p* = 0.004), a metastatic burden of fewer than two sites (HR: 0.248; 95% CI: 0.078–0.788; *p* = 0.018), and absence of recent antibiotic exposure (HR: 0.209; 95% CI: 0.064–0.687; *p* = 0.010) remained independent predictors of prolonged PFS (Table 3). RAS status did not retain independent prognostic significance in the multivariate model and was therefore not included in the final PFS model.

When survival outcomes were analyzed according to primary tumor location, no statistically significant differences in progression-free survival or overall survival were observed between patients with right-sided and left-sided disease. Although numerically longer survival was observed in patients with right-sided tumors, these differences were not statistically significant, and the wide confidence intervals of the hazard ratios preclude any reliable inference (Table S2).

### 3.3. Treatment Modality and Survival

**Figure 3:**
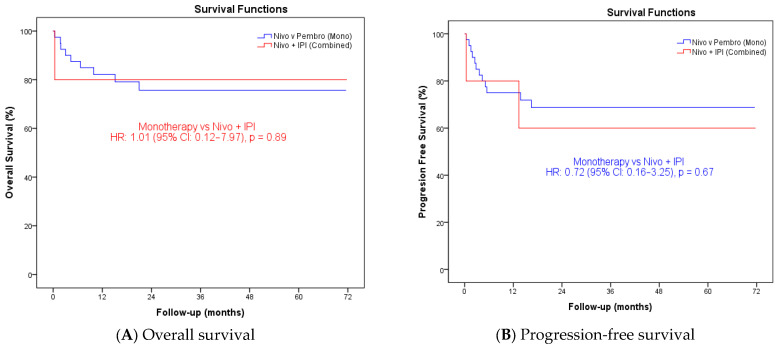
Kaplan–Meier Estimates of Overall and Progression-Free Survival by Immunotherapy Strategy. Kaplan–Meier survival curves comparing patients treated with immune checkpoint inhibitor monotherapy (nivolumab or pembrolizumab) and combination therapy with nivolumab plus ipilimumab. (**A**) Overall survival and (**B**) progression-free survival are shown. Abbreviations: HR, hazard ratio; CI, confidence interval; Nivo, nivolumab; IPI, ipilimumab.

When treatment modality was evaluated using Cox proportional hazards regression, no statistically significant differences in survival outcomes were observed between patients receiving immune checkpoint inhibitor monotherapy and those treated with nivolumab plus ipilimumab. With respect to overall survival, immune checkpoint inhibitor monotherapy was not associated with a significantly different risk of death compared with combination therapy (HR: 1.01, 95% CI: 0.13–7.98; *p* = 0.993). Similarly, progression-free survival did not differ significantly between the two treatment approaches, although a numerically lower risk of disease progression was noted in the monotherapy group (HR: 0.73, 95% CI: 0.16–3.25; *p* = 0.677). The wide confidence intervals observed in both analyses reflect the limited sample size and the small number of patients treated with combination immunotherapy (Figure 3).

### 3.4. Treatment Response and Subgroup Analyses

When treatment response was evaluated in relation to baseline characteristics, a lower disease burden, reflected by the presence of a single metastatic site, was associated with higher objective response and disease control rates (Table S1). In addition, the absence of peritoneal metastases was associated with more favorable response outcomes. No meaningful associations were identified between treatment response and immunotherapy regimen, sex, or liver metastases.

## 4. Discussion

In this real-world study, we assessed the effectiveness and tolerability of immune checkpoint inhibitors in patients with metastatic colorectal cancer characterized by MSI-H or dMMR. Our results were largely consistent with those reported in pivotal randomized trials. The 1-, 2-, and 3-year OS and PFS rates observed in our cohort indicate that durable clinical benefit can be achieved with immunotherapy in this molecularly defined population. Beyond survival outcomes, we also identified performance status, metastatic burden, and recent antibiotic exposure as independent prognostic factors for both OS and PFS. These findings highlight the importance of baseline patient condition, disease extent, and microbiome integrity in shaping immunotherapy outcomes, offering a practical framework for treatment selection in daily oncology practice [10,12].

Our findings are consistent with the results of the KEYNOTE-177 trial, which established the efficacy of pembrolizumab as first-line therapy in MSI-H/dMMR mCRC. In KEYNOTE-177, the median PFS in the pembrolizumab arm was 16.5 months, with approximately 30% of patients experiencing early disease progression. In our cohort, where 75.6% of patients received immunotherapy in the second line or beyond, a 3-year PFS rate of 70% was achieved. This suggests that the robust and durable benefits of immune checkpoint inhibition can be maintained even in real-world settings and later lines of therapy, highlighting the resilience of this treatment approach across diverse clinical scenarios, although small sample size and possible selection bias as the factors contributing to low rates of early progression cannot be excluded [10].

In one of the largest real-world series to date, the AGEO study reported a median PFS of 37.9 months in patients with advanced MSI-H/dMMR colorectal cancer treated with immune checkpoint inhibitors, accompanied by remarkably high long-term survival rates. Despite the relatively limited sample size of our cohort, the 3-year PFS rate of 67.5% observed in our analysis aligns closely with these results, supporting the notion of sustained long-term clinical benefit. This concordance with multicenter data from the AGEO study further underscores the robust and durable efficacy of immunotherapy across diverse patient populations [15].

These observations are also consistent with the results of the phase II KEYNOTE-158 trial, which evaluated pembrolizumab in previously treated MSI-H or dMMR advanced solid tumors, including a substantial cohort of patients with metastatic colorectal cancer. In that study, the objective response rate was 33% in the metastatic colorectal cancer subgroup, with many responses lasting beyond two years, and the median duration of response was not reached at the time of the primary analysis. Importantly, updated long-term follow-up data from KEYNOTE-016 presented at the ASCO 2025 Annual Meeting demonstrated a 10-year overall survival rate of 47%, supporting the concept of durable long-term disease control and suggesting the possibility of cure in a subset of patients. In this context, the sustained survival and long-term disease control observed in our real-world cohort, despite most patients receiving immune checkpoint inhibitors in later lines of therapy, are consistent with these findings and further underscore the capacity of immunotherapy to provide prolonged benefit in this biomarker-defined population [4,16].

Similarly, in a real-world analysis from the MD Anderson Cancer Center, the median OS in patients with advanced MSI-H/dMMR colorectal cancer treated with immunotherapy was reported as 49.1 months, with a 12-month OS rate of 88.2% [17]. These findings reinforce that the robust and durable efficacy of immunotherapy extends beyond controlled clinical trial settings to routine clinical practice. Likewise, in our cohort, the 36-month OS rate of 76.7% indicates that this benefit can be maintained across different healthcare systems and clinical infrastructures [18].

The CheckMate-142 trial, a multicenter phase II study, demonstrated that nivolumab alone or combined with low-dose ipilimumab yielded durable clinical benefit in MSI-H/dMMR mCRC, with objective responses exceeding 50% and median PFS and OS not reached at extended follow-up [12]. These results are concordant with our real-world findings, where long-term survival was preserved despite most patients receiving ICIs beyond the first line. Collectively, our data extend the evidence from CheckMate-142 into routine clinical practice, reinforcing the robust activity of immunotherapy in MSI-H/dMMR mCRC [19].

Recent phase III evidence from the CheckMate 8HW trial demonstrated a statistically significant improvement in progression-free survival with nivolumab plus ipilimumab compared with nivolumab monotherapy in patients with dMMR metastatic colorectal cancer, including individuals previously treated with systemic therapy. However, no overall survival benefit has been established for the doublet regimen to date. Moreover, analyses restricted to the first-line population presented at ESMO did not meet statistical significance for progression-free survival benefit [20]. In the present real-world cohort, the number of patients treated with combination immunotherapy was limited, and the study was not powered to evaluate comparative efficacy between immunotherapy regimens. Accordingly, no conclusions regarding the relative effectiveness of monotherapy versus combination therapy can be drawn from our data. Within this context, our results should be viewed as descriptive and complementary to randomized trial evidence, reflecting real-world clinical practice rather than comparative treatment effects.

In our cohort, a higher metastatic burden was strongly associated with poorer treatment response, while the absence of peritoneal metastases showed a borderline association with higher objective response and disease control rates (Table S1). These findings underscore the importance of tumor burden and metastatic distribution in shaping immunotherapy efficacy. Overall, the ORR and DCR observed in our study are consistent with results from pivotal trials that established immune checkpoint inhibitors as the standard of care for MSI-H/dMMR metastatic colorectal cancer, including KEYNOTE-177, CheckMate-142, and CheckMate-8HW. Response patterns across different immunotherapy regimens in our cohort closely mirrored those reported in these studies, with favorable outcomes observed for both pembrolizumab monotherapy and nivolumab-based regimens. Notably, reduced response rates among patients with RAS-mutant tumors and liver metastases parallel the attenuated efficacy described in corresponding subgroups of CheckMate-8HW, suggesting that biologically driven resistance patterns identified in clinical trials are also evident in real-world practice [10,12,20]. Taken together, these real-world findings closely reflect the biologically driven response patterns observed in pivotal clinical trials and highlight the central role of tumor burden, metastatic distribution, and molecular profile in shaping immunotherapy outcomes in MSI-H/dMMR metastatic colorectal cancer [21,22].

In addition to tumor and treatment related prognostic factors, increasing attention has been directed toward host related biomarkers that may provide complementary prognostic information in colorectal cancer. Emerging evidence suggests that serum cholinesterase, particularly butyrylcholinesterase (BChE), may serve as a prognostic biomarker in this setting. A recent study showed that low cholinesterase levels were associated with significantly worse survival outcomes, even among patients with tumor marker negative colorectal cancer, indicating that BChE may reflect systemic disease burden and host related factors rather than tumor burden alone. Reduced BChE levels have been linked to cancer-related inflammation, nutritional impairment, and metabolic dysfunction, all of which are known contributors to poor prognosis [23]. Although BChE was not evaluated in our cohort, these findings suggest that incorporating cholinesterase measurements into future prospective studies may provide additional prognostic value and help refine risk stratification in colorectal cancer patients.

One of the most striking findings of our study was the significant detrimental impact of antibiotic use within one month prior to ICI initiation on both OS and PFS. This observation is consistent with preclinical and clinical evidence suggesting that antibiotic-induced alterations in the gut microbiota may attenuate the immunomodulatory effects of ICIs [24,25]. In addition, having an ECOG performance status ≥1 and the presence of ≥2 metastatic sites emerged as independent prognostic factors for OS, consistent with previous reports [26]. Notably, peritoneal metastases have also been associated with lower response rates to immunotherapy in dMMR CRC [27]. However, due to the low number of cases, we were unable to draw definitive conclusions from this analysis. One of the major strengths of our study is its real-world design, encompassing patient populations frequently encountered in daily oncology practice but often underrepresented in randomized clinical trials. This enhances the external validity and applicability of our findings to broader clinical settings. Nevertheless, certain limitations must be acknowledged. The retrospective nature of the analysis introduces the possibility of underreporting adverse events and the potential for selection bias. Furthermore, the small sample size prevented us from conducting additional analyses for prognostic factors and from comparing ICI monotherapy with ICI–ICI combinations. Lastly, most patients were treated in later lines of therapy, limiting generalizability to high-resource settings [28].

## 5. Conclusions

This multi-center study provides the first real-world evidence from Türkiye evaluating the clinical outcomes and prognostic factors associated with immune checkpoint inhibitor therapy in patients with MSI-H/dMMR metastatic colorectal cancer. Despite the majority of patients receiving immunotherapy in later treatment lines, survival outcomes were largely consistent with those reported in pivotal phase III trials, supporting the robust and durable efficacy of immunotherapy in this molecularly defined subgroup across heterogeneous real-world settings. The multi-center design further enhances the generalizability of these findings by reflecting routine clinical practice across different oncology centers. Overall, our results demonstrate the sustained clinical benefit of immunotherapy and highlight the importance of prospective real-world registries and large collaborative efforts to further refine patient selection, integrate biomarker-driven stratification, and optimize the timing of immunotherapy in MSI-H/dMMR colorectal cancer.

## Figures and Tables

**Table 1:** Baseline Demographic and Clinical Characteristics of Patients Receiving Immunotherapy (N = 45).

Characteristic	N (%) or Mean ± SD
Age	
Median (range)	61 (21–78)
<65 years	26 (57.8%)
≥65 years	19 (42.2%)
Sex	
Male	33 (73.3%)
Female	12 (26.7%)
ECOG Performance Status	
0	28 (62.2%)
1	12 (26.7%)
≥2	5 (11.1%)
Primary tumor location	
Right colon	29 (64.4%)
Left colon	9 (20.0%)
Rectum	7 (15.6%)
TNM Stage at Diagnosis	
Stage I	1 (2.2%)
Stage II	5 (11.1%)
Stage III	16 (35.6%)
Stage IV	23 (51.1%)
Liver metastasis	
Yes	28 (60.0%)
No	17 (40.0%)
Lung metastasis	
Yes	13 (28.9%)
No	32 (71.1%)
Only liver metastasis	
Yes	14 (31.1%)
No	31 (68.9%)
Peritoneal metastasis	
Yes	15 (33.3%)
No	30 (66.7%)
Number of metastatic sites	
≤1	26 (57.8%)
>1	19 (42.2%)
Family history of colorectal cancer	
No	34 (75.6%)
Yes	8 (17.8%)
Unknown	3 (6.7%)
Method of MSI detection	
IHC	39 (86.7%)
PCR	1 (2.2%)
Both	3 (6.6%)
NGS	2 (4.4%)
BRAF mutation status *	
BRAF mutant	12 (26.7%)
BRAF wild-type	29 (64.4%)
RAS mutation status *	
KRAS mutant	8 (17.8%)
NRAS mutant	1 (2.2%)
RAS wild-type	33 (73.3%)
Unknown	3 (6.7%)
Antibiotic use within 1 month prior to ICIs	
Yes	7 (15.6%)
No	38 (84.4%)
Type of immunotherapy	
Pembrolizumab	33 (73.3%)
Nivolumab	7 (15.6%)
Nivolumab + Ipilimumab	5 (11.1%)
Immunotherapy treatment lines	
1	11 (24.4%)
2	16 (35.6%)
3	12 (26.7%)
4	6 (13.3%)

Abbreviations: BRAF, v-Raf murine sarcoma viral oncogene homolog B1; KRAS, Kirsten rat sarcoma viral oncogene homolog; NRAS, neuroblastoma RAS viral oncogene homolog; ECOG, Eastern Cooperative Oncology Group; TNM, Tumor-Node-Metastasis; MSI, Microsatellite instability; RAS, Rat sarcoma viral oncogene homolog; ICIs, Immune checkpoint inhibitors; IHC: Immunohistochemistry; PCR: Polymerase Chain Reaction; NGS: Next-generation sequencing. -* RAS and BRAF analysis available for 41 patients.

**Table 2:** Best response to immunotherapy in the study cohort (N = 45).

Variable	n	%
Overall Response	27	60
Best Response Category		
Complete response (CR)	7	15.6
Partial response (PR)	20	44.4
Stable disease (SD)	5	11.1
Progressive disease (PD)	11	24.4
Hyperprogressive disease (HPD) ^1^	2	4.4
Total	45	100

^1^ HPD definition: Progression within 2 months after treatment initiation accompanied by ≥50% increase in tumor burden. Objective response rate (ORR) = CR + PR = 60.0%. Disease control rate (DCR) = CR + PR + SD = 71.1%.

**Table 3:** Multivariate cox regression analyses for progression-free survival and overall survival.

		PFS			OS	
Variable	HR	95% CI	*p*	HR	95% CI	*p*
ECOG status (<1 vs. ≥1)	0.172	0.052–0.573	0.004	0.072	0.012–0.453	0.005
Number of metastases (≤1 vs. >1)	0.248	0.078–0.788	0.018	0.211	0.052–0.860	0.030
Antibiotic within 1 month pre-ICI (no vs. yes)	0.209	0.064–0.687	0.010	0.145	0.034–0.614	0.009

Abbreviations: OS, overall survival; PFS, progression-free survival; HR, hazard ratio; ICI, immune checkpoint inhibitor; ECOG Eastern Cooperative Oncology Group.

## Data Availability

The data that supports the findings of this study are available in the manuscript.

## Supplementary Materials

The following supporting information can be downloaded at: https://www.mdpi.com/article/10.3390/jcm15041554/s1, Table S1: Association of baseline clinicopathological characteristics with objective response rate (ORR) and disease control rate (DCR); Table S2: Univariate Cox regression analyses for progression-free survival and overall survival.

## Abbreviations

The following abbreviations are used in this manuscript:

BRAF	V-Raf murine sarcoma viral oncogene homolog B1
CI	Confidence interval
CR	Complete response
DCR	Disease control rate
ECOG	Eastern Cooperative Oncology Group
HR	Hazard ratio
ICI	Immune checkpoint inhibitor
IHC	Immunohistochemistry
KRAS	Kirsten rat sarcoma viral oncogene homolog
NGS	Next-generation sequencing
NRAS	Neuroblastoma RAS viral oncogene homolog
ORR	Objective response rate
OS	Overall survival
PCR	Polymerase Chain Reaction
PD	Progressive disease
PFS	Progression-free survival
PR	Partial response
SD	Stable disease
