# Supplementary material for: Efficacy, Safety, and Survival Outcomes of Immune Checkpoint Inhibitors in Patients with Mismatch Repair-Deficient Colorectal Cancer: A Retrospective, Multicenter Study
Source: J Clin Med. 2026 Feb 16;15(4):1554. doi: 10.3390/jcm15041554 (PMC12942511; doi:10.3390/jcm15041554)
Supplement: Supplementary file 1 [file jcm-15-01554-s001.zip › jcm-4130846-supplementary.pdf]

**Supplementary Material**

**Table S1.** Association of baseline clinicopathological characteristics with objective response rate (ORR) and disease control rate (DCR)

Values are presented as n (%).

| Variable | ORR Present | ORR Absent | ORR p-value | Disease Controlled | Disease Not Controlled | DCR p-value |
|---|---|---|---|---|---|---|
| **Sex** | | | 0.735 | | | 1.000 |
| Female | 8 (66.7%) | 4 (33.3%) | | 9 (75.0%) | 3 (25.0%) | |
| Male | 19 (57.6%) | 14 (42.4%) | | 23 (69.7%) | 10 (30.3%) | |
| **Metastatic Sites** | | | 0.013 | | | 0.008 |
| Single site | 21 (75.0%) | 7 (25.0%) | | 24 (85.7%) | 4 (14.3%) | |
| >1 site | 6 (35.3%) | 11 (64.7%) | | 8 (47.1%) | 9 (52.9%) | |
| **Liver Metastases** | | | 0.222 | | | 0.188 |
| Present | 14 (51.9%) | 13 (48.1%) | | 17 (63.0%) | 10 (37.0%) | |
| Absent | 13 (72.2%) | 5 (27.8%) | | 15 (83.3%) | 3 (16.7%) | |
| **Only Liver Mets** | | | 0.343 | | | 0.178 |
| Present | 10 (71.4%) | 4 (28.6%) | | 12 (85.7%) | 2 (14.3%) | |
| Absent | 17 (54.8%) | 14 (45.2%) | | 20 (64.5%) | 11 (35.5%) | |
| **Peritoneal Mets** | | | 0.105 | | | 0.086 |
| Present | 6 (40.0%) | 9 (60.0%) | | 8 (53.3%) | 7 (46.7%) | |
| Absent | 21 (70.0%) | 9 (30.0%) | | 24 (80.0%) | 6 (20.0%) | |
| **RAS Status** | | | 0.124 | | | 0.090 |
| Wild-type | 22 (66.7%) | 11 (33.3%) | | 26 (78.8%) | 7 (21.2%) | |
| Mutant | 3 (33.3%) | 6 (66.7%) | | 4 (44.4%) | 5 (55.6%) | |
| **BRAF Status** | | | 0.084 | | | 0.457 |
| Wild-type | 15 (51.7%) | 14 (48.3%) | | 20 (69.0%) | 9 (31.0%) | |
| Mutant | 10 (83.3%) | 2 (16.7%) | | 10 (83.3%) | 2 (16.7%) | |
| **Immunotherapy Regimen** | | | 0.986 | | | 0.638 |
| Pembrolizumab | 20 (60.6%) | 13 (39.4%) | | 24 (72.7%) | 9 (27.3%) | |
| Nivolumab | 4 (57.1%) | 3 (42.9%) | | 4 (57.1%) | 3 (42.9%) | |
| Nivo + Ipi | 3 (60.0%) | 2 (40.0%) | | 4 (80.0%) | 1 (20.0%) | |

*Disease control was defined as the achievement of complete response, partial response, or stable disease*

**Table S2.** Univariate cox regression analyses for progression-free survival and overall survival.

| | PFS | | | OS | | |
|---|---|---|---|---|---|---|
| Variable | HR | 95% CI | p | HR | 95% CI | p |
| Age (<65 vs. ≥65) | 0.672 | 0.235–1.919 | 0.458 | 0.678 | 0.196–2.346 | 0.540 |
| Sex (men vs. women) | 0.714 | 0.199–2.567 | 0.606 | 0.674 | 0.143–3.185 | 0.618 |
| ECOG status (<1 vs. ≥1) | 0.193 | 0.063–0.595 | 0.004 | 0.079 | 0.016–0.398 | 0.002 |
| Tumor location: Right vs Left colon | 0.692 | 0.240–1.997 | 0.496 | 0.515 | 0.149–1.782 | 0.295 |
| Lung metastases (no vs yes) | 0.737 | 0.247–2.201 | 0.585 | 0.968 | 0.250–3.743 | 0.962 |
| Liver metastases (no vs yes) | 0.385 | 0.107–1.381 | 0.143 | 0.336 | 0.071–1.587 | 0.169 |
| Only Liver metastases (yes vs no) | 0.528 | 0.147–1.895 | 0.327 | 0.514 | 0.109–2.420 | 0.399 |
| Peritoneal metastases (no vs yes) | 0.383 | 0.132–1.107 | 0.076 | 0.261 | 0.073–0.936 | 0.049 |
| Number of metastases (≤1 vs >1) | 0.291 | 0.097–0.870 | 0.027 | 0.238 | 0.062–0.923 | 0.038 |
| BRAF, KRAS, and NRAS all wild-type | 0.602 | 0.190–1.905 | 0.338 | 0.479 | 0.114–2.016 | 0.315 |
| BRAF mutant vs wild | 2.418 | 0.598–9.065 | 0.255 | 1.377 | 0.277–6.844 | 0.696 |
| KRAS/NRAS wild vs mutant | 0.162 | 0.048–0.550 | 0.034 | 0.289 | 0.042–0.761 | 0.048 |
| Antibiotic within 1 month pre-ICI (no vs yes) | 0.125 | 0.040–0.390 | 0.001 | 0.073 | 0.019–0.277 | 0.001 |

Abbreviations: BRAF, v-Raf murine sarcoma viral oncogene homolog B1; KRAS, Kirsten rat sarcoma viral oncogene homolog; NRAS, neuroblastoma RAS viral oncogene homolog; ECOG-PS, Eastern Cooperative Oncology Group performance status; HR, hazard ratio; CI, confidence interval; NE, not estimable; OS, overall survival; PFS, progression-free survival; ICI, immune checkpoint inhibitor,
